# Development and Experimental Validation of an Integrated Evaluation Framework for EMS Smartwear Electrodes

**DOI:** 10.3390/s25247484

**Published:** 2025-12-09

**Authors:** Gihyun Lee, Uri Chae, Jungmin Yun, Donghyeon Seo, Inyoung Jang, Geunho Ham, Taehoon Kim, Hongbum Kim

**Affiliations:** 1 Korea National Industrial Convergence Center, Korea Institute of Industrial Technology, Ansan 15588, Republic of Korea; ghlee@kitech.re.kr (G.L.); jmyun@kitech.re.kr (J.Y.); dhseo@kitech.re.kr (D.S.); 2 Innovative Transportation and Logistics Research Center, Korea Railroad Research Institute, Uiwang 16105, Republic of Korea; chaeuri@krri.re.kr; 3 Corporate R&D Center, HOSPI, Hanam 12991, Republic of Korea; inyoung.jang@hospi.co.kr (I.J.); geunho.ham@hospi.co.kr (G.H.); 4 College of Business, Gachon University, Seongnam 13120, Republic of Korea

**Keywords:** smartwear, EMS, electrical muscle stimulation, evaluation framework, muscle fatigue, electromyography, tensiomyography, user feedback

## Abstract

This study presents an integrated evaluation framework for textile-based electrical muscle stimulation smartwear electrodes, combining physiological and user-centered assessments to ensure comprehensive performance analysis. Four electrode types—lock stitch, knit, hot stamping, and moss stitch—were examined using a systematic five-step process with nine participants. Quantitative measurements were obtained using electromyography to determine maximum voluntary contraction and tensiomyography to assess muscle contraction velocity. The knit electrode demonstrated a statistically significant reduction in maximum voluntary contraction following stimulation (W = 2.0, *p* = 0.012, Cohen’s d = 0.58), indicating effective neuromuscular activation and fatigue induction. The moss stitch electrode also showed a notable trend toward reduced muscle activation (W = 6.0, *p* = 0.055, d = 0.37). In contrast, the lock stitch and hot stamping electrodes exhibited negligible changes. User experience surveys revealed overall high acceptance across all electrode types (4.0–4.5 of mean scores on a 5-point scale), with the moss stitch electrode receiving the highest ratings for perceived safety and minimal skin discomfort, while the hot stamping electrode scored lowest in breathability. The proposed framework enables balanced evaluation of both functional performance and user experience, offering practical design guidance for optimizing textile electrodes across applications ranging from high-intensity athletic training to low-intensity rehabilitation.

## 1. Introduction

Interest in smart wearable electronic systems has grown rapidly in the healthcare and fitness sectors [1]. While conventional wearable devices have primarily been used for monitoring physiological signals, recent advances have focused on integrating electrical muscle stimulation (EMS) into clothing for diverse applications, including exercise training, rehabilitation therapy, and haptic feedback in virtual reality environments [2,3,4,5]. For instance, EMS technology has been applied not only to full-body suits for muscle strengthening and rehabilitation but also to smart garments that provide tactile feedback in virtual environments by directly stimulating the wearer’s muscles to create novel sensory experiences [4]. Consequently, EMS-based smartwear has expanded from medical rehabilitation to sports training and augmented/virtual reality (AR/VR) applications, spurring extensive research and development in this emerging field [6,7].

Despite these advances, several technical challenges remain in implementing EMS in smartwear form [8]. One major limitation arises from the use of conventional gel electrodes. Although adhesive gel electrodes provide reliable conductivity and intimate skin contact, prolonged use leads to gel drying, which increases contact impedance, reduces stimulation efficiency, and causes adverse skin reactions such as irritation or allergies. Furthermore, gel electrodes are disposable, difficult to clean or reuse, and may cause discomfort owing to their direct adhesion to the skin, raising concerns about hygiene and long-term wearability [9].

To address these limitations, textile-based dry electrodes have recently gained significant attention [10]. Various fabrication techniques are being explored, including the integration of conductive fibers or metallic threads into fabrics and the application of conductive inks through printing processes [11,12,13]. Among these, embroidered textile electrodes exhibit excellent skin conformity and mechanical durability [14], whereas knit and hot-stamped electrodes offer advantages such as elasticity, breathability, and manufacturing simplicity, enhancing their potential as viable EMS smartwear electrodes [15].

Although numerous types of dry electrodes have been developed, a review of existing research reveals significant limitations in assessing the effectiveness and usability of textile electrodes. Most previous studies have focused on a single evaluation aspect—either objective indicators, such as electrical properties and stimulation performance, or subjective indicators, such as user comfort and wearability related to specific materials. For example, some studies concentrated exclusively on engineering parameters, including electrode impedance, stimulation thresholds, and evoked muscle force [11,14,16,17], whereas others examined only user-perceived outcomes such as skin irritation, pain, and overall comfort [13,18]. This fragmented evaluation approach hinders a comprehensive understanding of electrode performance, and a standardized framework that integrates both quantitative and qualitative assessments remains lacking.

To address these limitations and improve both the validity and practical relevance of electrode evaluation, this study introduces an integrated evaluation framework that combines quantitative physiological data with qualitative user experience information. Electromyography (EMG) measures electrical activity during muscle contraction, enabling quantitative evaluation of neuromuscular activation and fatigue, while tensiomyography (TMG) non-invasively assesses mechanical muscle responses, providing valuable insight into neuromuscular reactivity. However, even electrodes demonstrating superior stimulation efficiency may face limited practical adoption if user-related factors such as comfort, adhesion, skin compatibility, and wearability are unsatisfactory. These subjective aspects can be effectively captured through structured questionnaires and user interviews, offering essential qualitative insights that cannot be conveyed using quantitative analysis alone.

In summary, the proposed integrated evaluation framework establishes a standardized approach for the systematic development and assessment of EMS smartwear electrodes. By balancing technical performance with user satisfaction, it provides a practical methodology that supports the advancement of high-performance, user-friendly EMS smartwear technologies.

## 2. Overview of the Integrated Evaluation Framework

**Figure 1:**
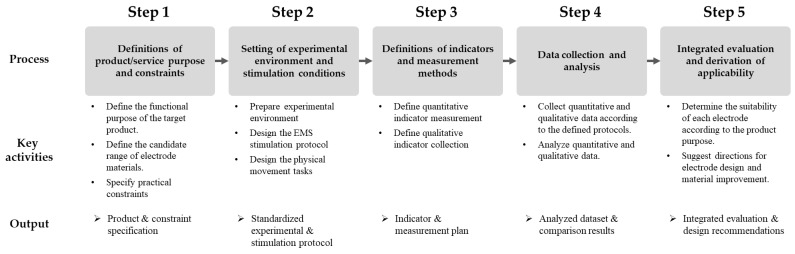
Step-by-step structure of the integrated evaluation framework.

The integrated evaluation framework for EMS smartwear electrodes is organized as a sequential process comprising five stages: (1) defining the purpose and operational constraints of the target product or service, (2) establishing the experimental environment and stimulation parameters, (3) specifying the performance indicators and corresponding measurement methods, (4) conducting data acquisition and analysis, and (5) performing integrated evaluation and determining overall applicability. The detailed structure of each stage is summarized in Figure 1.

The framework can be applied in the following contexts:

- Screening of textile electrodes: Enables the systematic comparison and evaluation of different candidate electrodes—such as embroidered, knitted, printed, and transferred types—to identify those most suitable for a given product or application.
- Design variable impact assessment: Evaluates the effect of design parameters, including electrode material and attachment position, on EMG (%MVC), TMG (Vc, Dm, Tc), wearability, and safety.
- Determination of application suitability: Integrates quantitative indicators (EMG, TMG) and qualitative indicators (user surveys and interviews) to identify the strengths, weaknesses, and practical applicability of each electrode type.

### 2.1. Definition of Product/Service Purpose and Constraints

The first stage involves defining the functional objectives of the product and identifying its practical constraints. This step serves as a foundation for determining the experimental design, selecting evaluation indicators, and setting performance criteria. It also provides the basis for adapting the framework to the specific requirements of different EMS applications.

During this stage, the intended function of the product (e.g., muscle strengthening or rehabilitation support) and its usage conditions (e.g., wearing duration or activity intensity) are clearly defined. Based on these parameters, the relevant performance indicators are selected and prioritized. This approach enables the characterization of electrode properties in relation to the intended purpose of the product, ensuring both consistency in experimental design and reliability in data interpretation.

### 2.2. Experimental Environment and Stimulation Condition Setup

To ensure reliable and reproducible comparisons among different electrode materials, this step follows a standardized procedure that establishes a consistent experimental environment and predefined stimulation conditions and performance tasks.

The experimental setup is designed to maintain controlled and uniform conditions by regulating external factors such as room temperature and humidity. The EMS controller, EMG/TMG measurement systems, evaluation chair, and TMG bed are arranged efficiently according to the subject’s movement path during testing. Standardized clothing, such as shorts, is provided to minimize sensor interference. Additionally, the subject’s movement route and task locations are prearranged to facilitate posture correction and ensure smooth, repeatable measurements.

The stimulation conditions are defined within a physiological range sufficient to induce muscle activation and include parameters such as frequency, pulse width, and contraction–rest cycles. Stimulation intensity is individually adjusted based on each subject’s sensory threshold and tolerance to discomfort, while the total stimulation duration is limited to prevent excessive muscle fatigue.

Evaluation tasks are designed to correspond to the targeted muscle group. For example, lower-limb electrodes activate thigh muscles through a static half-squat posture, as summarized in Table 1, whereas upper-limb electrodes may involve elbow flexion–extension or resistance exercises. All subjects receive prior training to ensure uniform posture and repetition across trials. During measurements, the experimenter continuously monitors and corrects posture to maintain consistency.

The proposed stimulation protocol and task design are independent of specific equipment or electrode configurations, enabling flexible application to different electrode materials and anatomical sites. This standardized approach enables consistent comparison of electrode performance under uniform conditions and supports adaptation for diverse muscle groups and evaluation objectives.

### 2.3. Definition of Collection Indicators and Measurement Methods

In this step, responses to EMS electrode stimulation are evaluated using two complementary categories of indicators: quantitative performance metrics and qualitative user experience measures. Together, these indicators provide a comprehensive assessment of both the stimulation efficiency and the perceived user satisfaction associated with each electrode type.

Quantitative indicators serve to objectively quantify neuromuscular responses and compare the performance of EMS electrodes. Within this framework, EMG and TMG are employed as the primary measurement tools. EMG assesses muscle electrical activity and determines the percentage of maximum voluntary contraction (MVC) before and after stimulation, relative to each participant’s baseline MVC. A significant decrease in %MVC after stimulation indicates muscle fatigue, whereas maintenance or an increase signifies enhanced neuromuscular activation. TMG evaluates mechanical contraction properties using three key parameters: maximum radial displacement (Dm), contraction time (Tc), and contraction velocity (Vc). A reduction in Vc may indicate fatigue or diminished responsiveness, while prolonged Tc and reduced Dm suggest muscle stiffness or fatigue. These indicators are measured under identical pre- and post-stimulation conditions to enable precise quantitative comparison of neuromuscular responses across different electrode materials.

Qualitative indicators complement the quantitative results by capturing subjective aspects of the user experience that cannot be expressed numerically. These are collected through structured questionnaires and semi-structured interviews. The questionnaire comprises eight items, listed in Table 2, addressing factors such as safety, comfort, and fabric texture. Each item is rated on a five-point Likert scale (1: strongly disagree to 5: strongly agree). Additionally, the interviews allow participants to freely discuss perceived differences among electrodes, sources of discomfort, and potential improvements, thereby revealing detailed insights into user perception and contextual preferences that quantitative data alone cannot convey.

### 2.4. Data Collection and Analysis

In this step, data are collected according to predefined quantitative and qualitative indicators, followed by statistical analyses to objectively identify performance differences among electrode materials. Quantitative and qualitative data are processed using distinct analytical methods and subsequently integrated to enable a comprehensive evaluation of electrode performance.

For quantitative indicators, EMG and TMG data are statistically analyzed to assess pre–post changes for each electrode type. Paired-sample tests are applied to each electrode material to evaluate variations before and after stimulation, while differences across electrode types are compared using descriptive statistics. Effect size calculations are conducted to interpret the practical magnitude of the observed effects.

For qualitative indicators, survey and interview data are analyzed using a multilayered approach. Survey responses are processed using descriptive statistics and nonparametric tests to identify differences in user satisfaction across electrode materials. Interview responses are examined through content analysis to extract major themes, quantify their frequency, and summarize key user perceptions associated with each electrode type.

### 2.5. Integrated Evaluation and Applicability

This step aims to comprehensively assess the characteristics and application-specific suitability of each electrode material by integrating both quantitative and qualitative findings. This integrated approach allows the identification of electrodes that best align with the intended design objectives while highlighting design aspects that require further improvement.

If quantitative analyses indicate that a specific electrode exhibits superior physiological performance (e.g., reduced %MVC or increased Vc), it may be regarded as having high stimulation efficiency. However, if qualitative findings simultaneously reveal discomfort, skin irritation, or other negative user experiences, such factors must be considered limitations to practical usability. Conversely, electrodes showing relatively lower quantitative performance but high user satisfaction may be preferable for applications such as rehabilitation, long-term wear, or low-intensity stimulation environments.

Therefore, the integrated evaluation interprets quantitative and qualitative results in a complementary manner, ensuring that conclusions are drawn not from a single metric but in accordance with the intended purpose and context of the product or service.

## 3. Materials and Methods

### 3.1. Participant Composition and Experimental Procedure

To verify the applicability and practicality of the proposed evaluation framework, experimental validation was conducted. During the experiments, both quantitative and qualitative responses to EMS smartwear electrode materials were collected and analyzed in accordance with the framework’s procedures to confirm its structural validity and feasibility.

A total of 10 healthy adults (7 males and 3 females; mean age) voluntarily participated in the study, comprising five EMS experts and five non-experts. The participants’ mean height was 170.2 ± 7.64 cm, weight was 67.86 ± 16.69 kg, thigh circumference was 52.7 ± 5.3 cm, skeletal muscle mass was 27.89 ± 8.68 kg, body fat mass was 17.62 ± 5.4 kg, and body mass index (BMI) was 23.14 ± 3.57 kg/m^2^. None of the participants had a history of neuromuscular or musculoskeletal disorders, and all exhibited normal skin conditions at the electrode attachment site (vastus lateralis of the thigh). Individuals with implanted electronic devices such as pacemakers or any contraindication to electrical stimulation therapy were excluded from participation.

All participants received full information regarding the study’s objectives and procedures before enrollment and voluntarily provided written informed consent. The study was conducted in compliance with ethical standards and was approved by the Institutional Review Board of the Korea Institute of Industrial Technology (KITECH; approval number A-2025-003).

The required minimum sample size was determined using G*Power software (version 3.1.9.7 [19]) based on the Wilcoxon signed-rank test (effect size dz = 1.0, α = 0.05, power = 0.80) [20], yielding a sample size of nine participants. To account for potential dropouts and outliers, ten participants were initially recruited, and data from nine participants were ultimately included in the analysis after excluding one outlier.

The experimental study was conducted over two days. During the first session, participants were briefed on the study procedures and provided written consent. To minimize the effects of muscle fatigue induced by EMS, the experiment was divided into two sessions. Before testing, participants completed a demographic questionnaire and underwent basic anthropometric measurements. On the first day, each participant was randomly assigned two electrode materials for evaluation, and the remaining two materials were assessed on the second day following the same procedure. The evaluation order was randomized for each participant to eliminate bias.

The testing procedure for each electrode material proceeded as follows. First, baseline neuromuscular activity and contraction characteristics were recorded by performing TMG measurements and MVC assessments using EMG, without the electrode attached to the thigh. The target electrode was then applied to the vastus lateralis, and the predefined EMS protocol (5 min) was executed.

Immediately after the stimulation phase, TMG and MVC measurements were repeated using the same procedure to obtain post-stimulation data. Following the completion of stimulation and measurement for each material, participants were given a 30–40 min rest and recovery period, during which they completed a questionnaire evaluating the comfort and usability of the tested electrode. After sufficient rest, the next material was tested following the same procedure. This sequence was performed for two materials on the first day and the remaining two on the second day, enabling each participant to evaluate all four electrode types.

### 3.2. Composition of Electrode Materials for Evaluation

**Figure 2:**
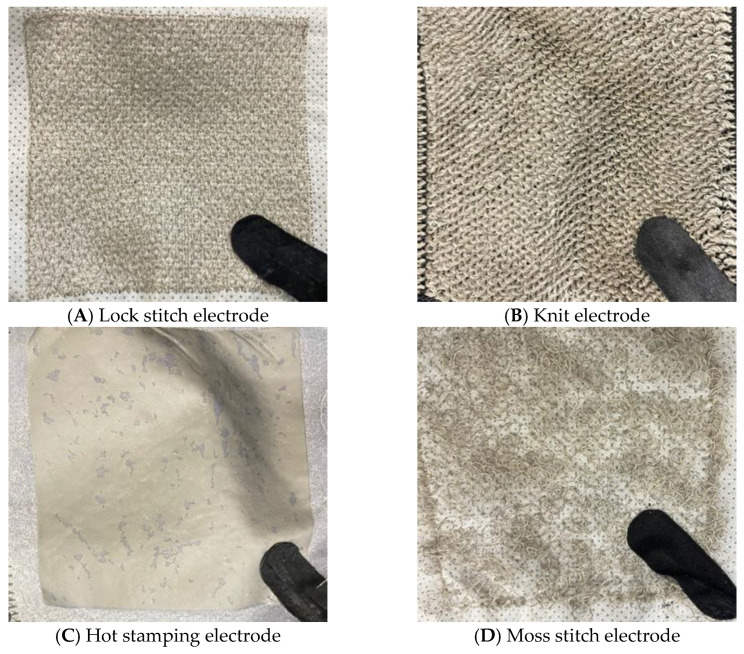
Four textile electrode materials selected for EMS performance evaluation.

As shown in Figure 2, four types of textile electrodes fabricated using different methods were selected for evaluation. Each electrode type differed in material structure, stimulation delivery characteristics, and wearability, allowing the identification of features best suited for the intended product application. Practically, these four electrode types represent fabrication techniques that are either widely studied or commonly employed in practical textile electrode applications. All electrodes were standardized to a square geometry (8 cm × 8 cm) to ensure consistent contact area. Silver-plated fibers or threads, known for their high electrical conductivity and antibacterial properties, were used as the electrode material. Each textile electrode was equipped with a magnetic snap-button connector on the reverse side for secure attachment to the EMS controller.

- Embroidery—lock stitch: Fabricated by inserting silver-plated polyamide conductive yarns (100 denier, 1 ply; Soitex, Goyang-si, Republic of Korea) into a non-woven polyester base fabric through a linear lock-stitch embroidery process using a computerized embroidery machine. The conductive yarns consist of polyamide filaments coated with a silver layer, providing high electrical conductivity (linear resistance: approximately 4.7 Ω/cm). The tightly interlaced conductive threads provide high structural stability and reproducibility.
- Knitting—knit: Produced by knitting silver-plated polyamide conductive yarns using a terry knit structure. The three-dimensional loop structure offers excellent elasticity, breathability, and enhanced skin contact while minimizing skin irritation during prolonged wear.
- Hot stamping: Manufactured by transferring a silver-based conductive sheet onto an elastic base fabric surface using a thermal transfer process. The conductive sheet consists of silver nanoparticles or silver flakes dispersed in a thermoplastic polyurethane (TPU) matrix, which serves as both the conductive layer and the adhesive binder. The transfer process is conducted under controlled conditions (temperature: 170 °C, pressure: 1 MPa, duration: 1 min) to ensure stable adhesion and electrical connectivity. This method enables direct electrode integration onto the elastic sleeve without additional sewing, allows the creation of complex patterns, and is well-suited for large-scale production.
- Embroidery—moss stitch: Constructed by vertically looping silver-plated polyamide conductive yarns (100 denier, 1 ply; Soitex, Goyang-si, Republic of Korea) onto a non-woven polyester base fabric using a moss stitch embroidery technique. The conductive yarns consist of polyamide filaments coated with a silver layer, providing high electrical conductivity (linear resistance: approximately 4.7 Ω/cm). The vertical loop structure forms a raised three-dimensional texture, resulting in a flexible electrode structure that provides a soft tactile sensation and enhanced conformability during skin contact.

All textile electrodes were integrated into identical elastic sleeves to ensure consistent application across participants, in accordance with the usage conditions defined during the product planning phase. The sleeves were fabricated using a knitted structure composed of nylon and polyester-based fabric blended with polyurethane-based elastic fibers, providing excellent stretchability, elasticity, and secure fit during prolonged wear. All sleeves were constructed with uniform dimensions and material composition to minimize variability in electrode positioning during application. Each electrode was first fabricated according to its structural type: embroidery and hot-stamping electrodes were applied onto a base fabric, while the knitted electrode was produced through self-supporting knitted construction. All electrodes were then processed into finalized patch forms and sewn onto designated positions within the elastic sleeves. This attachment method ensured consistent electrode placement across repeated stimulation sessions. In particular, the sleeve was able to provide sufficient elasticity and conformity within the average thigh circumference range (52.7 ± 5.3 cm), regardless of gender-related body shape differences, allowing it to be applied to both male and female participants without the need for separate sizing.

### 3.3. Validation of the Integrated Evaluation Framework

#### 3.3.1. Definition of Product/Service Purpose and Constraints

In this experiment, the intended product application was defined as smartwear designed for muscle strengthening and training support. The focus was placed on identifying electrodes capable of inducing muscle activation through EMS and promoting fatigue accumulation within a short duration. Accordingly, stimulation intensity, cycle parameters, and task composition were configured to emphasize the assessment of fatigue-inducing potential and neuromuscular responsiveness. The experimental design, including usage conditions and constraints, was established based on the usage scenarios summarized in Table 3.

These conditions were defined under the assumption that the target product would deliver effective muscle stimulation over a short time in exercise or fitness environments. Consequently, long-term wear or daily activity use was excluded from the experimental scope.

#### 3.3.2. Experimental Environment and Stimulation Conditions

All experiments were performed in the indoor laboratory of EMS Training Academy Korea. Following Step 2 of the evaluation framework, the experimental space was organized to optimize workflow efficiency, with the EMS controller, EMG system, and TMG setup arranged along the task path. This configuration ensured smooth participant and researcher movement, enabling systematic and efficient evaluation.

To minimize interference between the electrodes and sensors attached to the thigh, participants were instructed to wear short training pants appropriately sized to their body type. Textile electrodes were applied without any skin preparation—such as cleaning or hair removal—to replicate typical real-world conditions in which users apply EMS devices directly to the skin. The laboratory environment was kept at a comfortable indoor temperature, and all tests were conducted under consistent, disturbance-free conditions.

**Figure 3:**
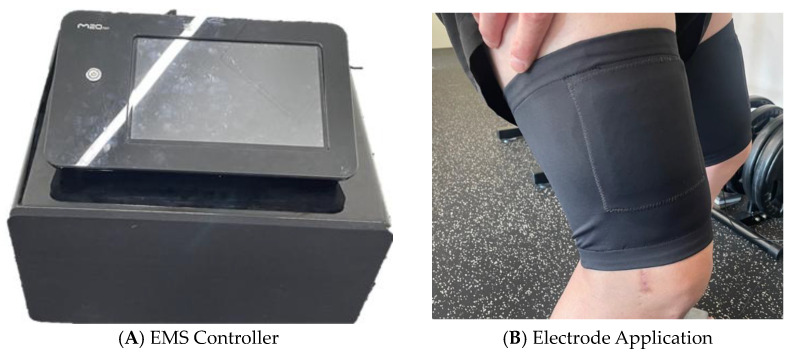
EMS controller and electrode application. (**A**) EMS controller allowed fine adjustment of stimulation parameters, including frequency, intensity, and duration, to enable precise and customized neuromuscular stimulation. (**B**) Application of electrodes to the target muscle site for accurate and effective delivery of electrical stimulation.

Precise control of EMS and repeatability of experimental conditions were ensured using a programmable EMS controller (M20 Co., Hanam-si, Republic of Korea), as shown in Figure 3. The device operates in both low-frequency (1–100 Hz) and medium-frequency (2000–6000 Hz) modes, with the low-frequency range utilized in this study for inducing muscle contraction and assessing fatigue. The controller enables fine adjustment of various stimulation parameters, including intensity (current output), contraction and relaxation duration, frequency modulation (increase/decrease), rest intervals, and mode switching. These parameters can be combined to construct individual stimulation programs (sequences), and multiple sequences can be integrated to implement customized stimulation protocols.

Electrical stimulation was administered through the EMS controller, configured to operate in a strength-training mode comprising 20 cycles of 8 s muscle contraction followed by 4 s relaxation. This protocol was selected based on commonly used EMS parameters for muscle strength enhancement and hypertrophy reported in prior research [21] and was designed to induce effective muscle activation and measurable fatigue within a short duration while reflecting the real-world usage scenario defined during the product planning phase. Before stimulation, participants performed a 60 s warm-up in a light half-squat position. The evaluation task required participants to maintain a static half-squat posture during the contraction phase and relax comfortably during the rest phase. This postural constraint was specifically designed to focus on the target muscle group (vastus lateralis), maximizing the synergistic effect of EMS and voluntary muscle contraction, thereby enabling consistent assessment of electrode neuromuscular responsiveness and fatigue-inducing potential across all electrode types. According to the experimental protocol described in Table 1, each electrode material was evaluated during approximately 5 min of EMS exercise.

The stimulation intensity was individually adjusted to each participant’s maximum tolerable level, considering skin sensations such as tingling or mild discomfort. This individualized approach was adopted because, while electrical stimulation requires sufficient intensity to elicit neuromuscular responses, excessive intensity can cause skin pain and discomfort that hinder experimental participation or reduce wearing compliance in real-world settings.

The intensity calibration procedure was conducted as follows: (1) participants assumed a half-squat position while wearing the textile electrode sleeve; (2) stimulation intensity was gradually increased from zero in increments of approximately 5 units; (3) participants verbally reported their sensory perception at each intensity level; and (4) the final intensity was set at the level where participants reported “tingling or mild discomfort but no pain,” ensuring effective muscle activation without excessive discomfort. This procedure was repeated for each electrode type. The EMS controller’s total output was fixed at 100 units, while the relative intensity applied to each electrode site was individually customized and recorded (Table 4). This approach ensured subjective equivalence of stimulation intensity across participants while allowing the assessment to focus on electrode material performance and user experience rather than absolute current levels. Furthermore, stimulation intensity was fine-tuned according to participant groups (P1: EMS experts; P2: non-experts) to ensure that all participants received effective yet comfortable stimulation levels.

#### 3.3.3. Collection Indicators and Measurement Methods

In this study, the performance of EMS electrodes was objectively evaluated using two validated physiological indicators: electrical muscle activity, measured using EMG, and mechanical muscle contraction characteristics, assessed using TMG. These methods have demonstrated high reliability and validity for assessing neuromuscular function and fatigue in prior research [22,23].

Electrical muscle activity was recorded using a wireless EMG system (MiniWave Infinity; Cometa Systems, Bareggio, Italy). Disposable surface electrodes were attached to the central belly of the vastus lateralis muscle, and the skin at the electrode site was cleaned with alcohol to reduce impedance, following standard surface electromyography guidelines (SENIAM). EMG signals were sampled at 2000 Hz.

To determine the MVC, participants were seated with joint movement restricted except for the target muscle and performed an isometric knee extension for approximately 5 s. This test was repeated three times, and the trial showing the highest EMG amplitude was selected as the MVC reference for subsequent %MVC calculations.

Mechanical contraction characteristics were measured using a TMG device (TMG-S2; TMG-BMC Ltd., Ljubljana, Slovenia). Participants lay on an examination bed, with their leg position stabilized using support pads to maintain a consistent posture during measurement. The TMG sensor was placed perpendicularly on the central belly of the vastus lateralis, and single-pulse electrical stimulation (1 ms) was applied to induce a contraction. The device recorded the corresponding surface displacement of the muscle. Both EMG and TMG measurements were conducted by trained evaluators following standardized procedures, and sensor attachment positions were kept consistent across all trials to ensure measurement reproducibility.

Subjective evaluation of electrode usability was conducted using a structured questionnaire comprising eight items, as summarized in Table 2, and the responses were converted into numerical scores for statistical analysis. To complement the questionnaire and obtain deeper qualitative insights, semi-structured interviews were also conducted. Participants were encouraged to freely discuss perceived differences among electrode types, preferred materials and their reasons, discomfort experienced, and potential areas for improvement. Example questions included “Which electrode did you find most comfortable, and why?” and “Did you experience any specific discomfort or issues with a particular material?”

#### 3.3.4. Data Analysis

To evaluate the effects of each textile electrode type, EMG and TMG data were statistically analyzed before and after EMS application. Due to the small sample size (n = 9), the Wilcoxon signed-rank test, denoted by W, was conducted to compare pre- and post-stimulation conditions. Effect sizes were calculated using Cohen’s *d* to determine the practical significance of observed differences, denoted by d. Differences across electrode types were compared using descriptive statistics, including means and standard deviations. Statistical significance was set at α = 0.05, and all analyses were performed using Python (version 3.13).

## 4. Results and Discussion

### 4.1. Results of Quantitative Indicator Data Analysis

#### 4.1.1. EMG MVC Measurement Results

**Figure 4:**
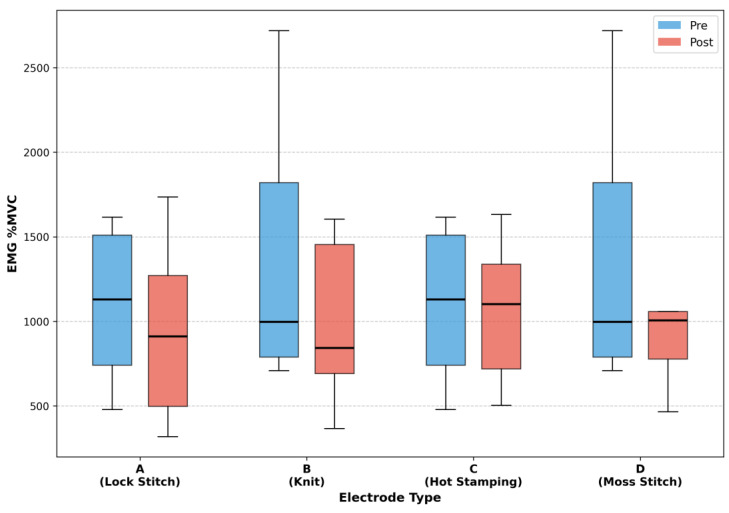
EMG %MVC before and after EMS application for each electrode type.

Figure 4 summarizes the comparison of muscle activity before and after electrical stimulation for each electrode material, using MVC as an indicator. Changes in MVC derived from EMG signals were analyzed to assess differences in neuromuscular stimulation efficiency among the electrode types. Statistical results for each electrode type are as follows: A (W = 13.0, *p* = 0.301, d = 0.19), B (W = 2.0, *p* = 0.012, d = 0.58), C (W = 21.0, *p* = 0.910, d = −0.08), and D (W = 6.0, *p* = 0.055, d = 0.37).

Material B (Knit) exhibited a statistically significant increase in MVC following stimulation, confirmed at the 5% significance level. This result indicates that the Knit electrode effectively promotes muscle activation and demonstrates relatively superior performance in terms of biocompatibility and stimulation delivery efficiency. Material D (Moss Stitch) also showed an increasing trend in MVC after stimulation, approaching statistical significance at the 10% level. Given the limited sample size, this finding suggests a potential enhancement in muscle activation attributable to electrical stimulation.

In contrast, Material A (Lock Stitch) and Material C (Hot Stamping) showed no statistically significant changes in MVC before and after stimulation. These results imply that their influence on muscle activation was relatively limited or that stimulation delivery under the given conditions was insufficient to induce a measurable response.

#### 4.1.2. TMG Vc Measurement Results

**Figure 5:**
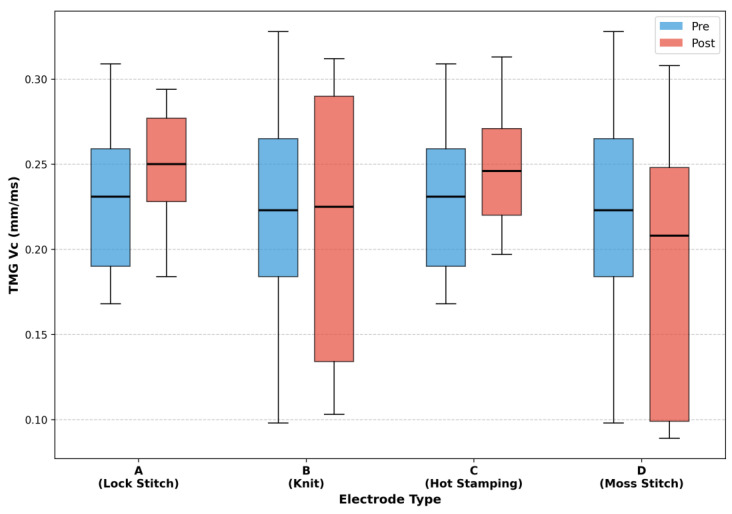
TMG Vc before and after EMS application for each electrode type.

Figure 5 presents the results of contraction velocity (Vc) measurements before and after stimulation, obtained using TMG. Among the four electrode types, only Material C (Hot Stamping) exhibited an increasing trend in Vc, with significance approaching the 10% level, suggesting a possible improvement in muscle contraction responsiveness. Statistical results for each electrode type are as follows: A (W = 11.0, *p* = 0.203, d = 0.49), B (W = 20.0, *p* = 0.820, d = −0.07), C (W = 8.0, *p* = 0.098, d = 0.61), and D (W = 12.0, *p* = 0.250, d = −0.50).

Material A (Lock Stitch) also showed a slight, non-significant increase in Vc, whereas Material D (Moss Stitch) exhibited a decreasing trend, indicating potential fatigue accumulation or less efficient stimulation transmission. Material B (Knit) maintained relatively stable Vc values before and after stimulation without notable variation.

Overall, these results demonstrate that each electrode material exerts distinct effects on neuromuscular response characteristics. In particular, Material C (Hot Stamping) appears to have a comparatively favorable influence on stimulation delivery efficiency, while Material B (Knit) exhibits superior capability for muscle activation.

### 4.2. Analysis of Qualitative Indicator Data

#### 4.2.1. Survey Results

**Figure 6:**
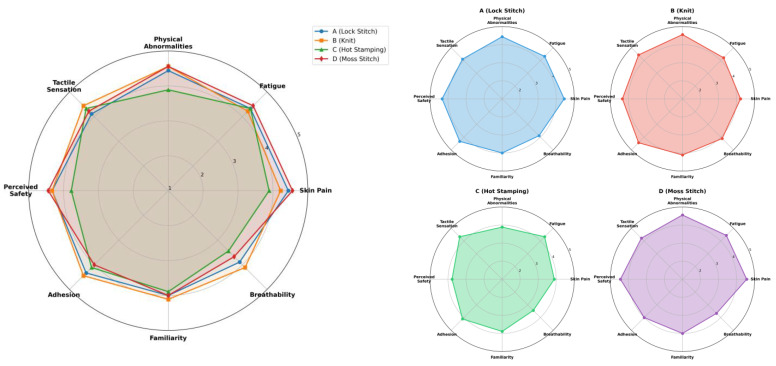
EMS controller and electrode.

The results of the participant survey on electrode usability are summarized in Figure 6. The average scores across the eight evaluation items revealed no substantial differences among the electrodes, although several material-specific characteristics were identified. Overall, all electrodes received favorable usability evaluations, with mean scores exceeding 4.0 on a five-point scale.

Material C (Hot Stamping) received comparatively lower ratings for skin pain, physical discomfort, and breathability, whereas Material D (Moss Stitch) demonstrated the most consistent and favorable responses in terms of skin adhesion and overall comfort.

#### 4.2.2. Interview Results

Participants also provided feedback regarding discomfort during wear and suggestions for improvement. The comments reflected diverse perspectives on factors such as fit, stimulation characteristics, adhesion, appearance, and general usability.

The key observations from the interviews can be summarized as follows. Material A (Lock Stitch) was described as providing gentle, massage-like stimulation; however, several participants noted insufficient depth of stimulation and occasional discomfort during wear. Material B (Knit) delivered relatively strong stimulation but was frequently reported to cause prickly sensations and inconsistent stimulation intensity. Material C (Hot Stamping) was perceived as producing minimal or imperceptible stimulation, leading some participants to question its functional effectiveness. Material D (Moss Stitch) was highly rated for soft wearing comfort, though concerns were expressed about its durability, visual appearance, adhesion consistency, and uneven stimulation distribution.

### 4.3. Integrated Analysis Results

In this study, electrode performance was comprehensively evaluated through an integrated approach combining quantitative physiological signal analysis with qualitative user feedback. Each electrode was assessed not only for changes in neuromuscular function before and after stimulation but also for subjective user experience, thereby enabling a balanced evaluation of functional effectiveness and user acceptability.

Quantitative EMG analysis of MVC revealed that Material B (Knit) produced a statistically significant increase in muscle activity, indicating the highest stimulation efficiency among the tested electrodes. This result suggests that the Knit electrode offers superior adhesion and electrical conductivity, facilitating efficient transmission of stimulation signals. This result can be used as further evidence of the effective transmission by knitted electrodes as investigated in [15,24]. Material D (Moss Stitch) also showed an upward trend in MVC, approaching statistical significance at the 10% level, implying potential for enhanced muscle activation. In contrast, Materials A (Lock Stitch) and C (Hot Stamping) exhibited no significant changes, indicating relatively stable performance across pre- and post-stimulation conditions.

For TMG-based contraction velocity (Vc), Material C (Hot Stamping) displayed an increasing trend at the 10% level, suggesting improved muscle contraction responsiveness. Material A (Lock Stitch) showed a non-significant upward tendency, whereas Material D (Moss Stitch) exhibited a decrease in Vc, indicating possible fatigue accumulation or suboptimal stimulation delivery. Material B (Knit), despite demonstrating strong EMG-based activation, showed minimal variation in Vc, suggesting that it may be more suitable for continuous or endurance-type stimulation rather than for rapid contraction responses.

The qualitative evaluation revealed similar distinctions among the electrode types. While survey results indicated generally high user acceptability, the subjective experiences of participants highlighted clear differences. The Knit electrode (Material B) delivered strong stimulation but elicited complaints regarding skin irritation and inadequate adhesion. This result is consistent with [18], which showed that textile electrodes can create hot spots of high current density. The Moss Stitch electrode (Material D), despite its limited stimulation effectiveness, provided superior comfort and wearability, suggesting suitability for low-intensity applications such as rehabilitation where user comfort shows a top priority [13]. The Hot Stamping electrode (Material C) was frequently criticized for weak stimulation and low overall acceptability, indicating a need for redesign to improve conductivity and stimulation delivery. The Lock Stitch electrode (Material A) was regarded as relatively well-balanced but lacking sufficient stimulation depth, suggesting its suitability for less intense or basic monitoring applications [14].

## 5. Conclusions

This study developed an integrated evaluation framework that simultaneously incorporates both performance metrics and user experience in assessing EMS smartwear electrodes and verified its practicality through experimental validation. Previous studies were limited to focusing on electrodes performance at an early experimental stage, requiring a parameter-centric measurements approach [11,14,15,17]. Furthermore, to overcome the limitation of a small number of participants, efforts were made to investigate user perception based only on the experiment result [13,18]. However, this also demonstrated limitations in collecting the various perspectives that could be derived through human subjective. To address these issues, the present framework combines physiological measurements using EMG and TMG with user responses obtained from structured surveys and interviews, enabling a multidimensional characterization of electrode performance by material type. The findings from this approach showed consistency with the results of previous studies, thus providing evidence for the usefulness of the integrated evaluation framework. In addition, these results indicate that each electrode type possesses distinct advantages depending on its intended application—ranging from high-intensity training to rehabilitation or long-duration, low-intensity use.

The proposed integrated evaluation framework allows systematic and consistent assessment of both technical performance and user acceptance across various electrode materials, thereby offering scientific guidance for product design and material optimization. Furthermore, the framework can be flexibly adapted to diverse conditions, including different target muscle groups, wearing environments, and user populations, demonstrating its potential applicability across rehabilitation devices, sports wearables, and personal fitness systems.

## 6. Limitations and Future Research

This study conducted an initial validation of the proposed EMS smartwear electrode evaluation framework with a sample of nine participants (seven males and two females). Although the sample size satisfied the minimum requirement for statistical power based on G*Power analysis, it remains limited for detecting sex-based differences in electrode performance. Physiological factors such as thigh circumference, subcutaneous fat thickness, and muscle density may differ between sexes and could influence electrode fit and stimulation delivery efficiency. Future studies should employ larger and more balanced samples to enable sex-stratified analyses.

Additionally, to reflect real-world usage conditions, electrodes were applied without skin preparation, and participants were not required to remove leg hair. Consequently, individual differences in hair density may have affected electrode–skin impedance. Future research should systematically examine the effect of hair density on electrode performance and consider standardized hair assessment or removal protocols to enhance measurement reliability.

Furthermore, comparing the textile electrodes used in this study with commercially available disposable gel electrodes would provide valuable insights into their relative performance and practical applicability as alternatives.

Finally, relatively large standard deviations were observed in some measurements, which may limit interpretability. This variability is in line with prior EMS studies that report considerable inter-individual differences due to physiological diversity, sensory thresholds, and personalized stimulation intensities [25,26]. Despite this, statistically significant differences were still identified between electrode types, suggesting that the proposed electrode designs have a meaningful effect on stimulation delivery. Future studies may benefit from more controlled participant stratification and multilayered analyses that account for user-specific characteristics.

Despite the limitations, this study corroborated the potential applicability of the integrated evaluation framework. Future research should extend this framework to evaluate long-term usability, durability under repeated use, and hygiene performance of different materials. Additionally, integrating adaptive control mechanisms, such as automatic intensity modulation and feedback-driven personalized stimulation algorithms, can further enhance the precision and practicality of EMS smartwear evaluation for next-generation applications.

## Figures and Tables

**Table 1:** Electrical stimulation protocol and sensor usability evaluation procedure.

Manual	Warm-Up	Exercise	Rest	Repetition
Muscle strengthening (5 min)	60 s	8 s	4 s	20 repetitions
	Individual intensity adjustment	Active phase	Rest phase	-
	Half squat and comfortable position after intensity adjustment	Half squat	Comfortable phase	

**Table 2:** Survey items used for assessing user experience.

No.	Evaluation Item (Survey Questions)	Scale and Response Criteria
1	Did you experience no pain on the skin while wearing this material?	1 (Strongly disagree)–5 (Strongly agree)
2	Did you feel no fatigue while wearing this material?	
3	Did you experience no physical abnormalities (e.g., skin changes, pain, swelling) during or after using this material?	
4	Was the sensation of this material on your skin satisfactory?	
5	Do you consider this material safe with respect to potential effects such as electromagnetic waves or heat on the human body?	
6	Did this material adhere well to the skin without detaching easily?	
7	Did wearing this garment feel natural and familiar rather than awkward?	
8	Even when worn for an extended period, did the garment allow heat and sweat to dissipate easily, maintaining comfort?	

**Table 3:** Summary of usage scenarios serving as the basis for experimental design.

Category	Setting Description
Wearing time	Approximately 5 min of intensive stimulation per session
Wearing environment	Indoor static exercise environment (e.g., maintaining a half-squat posture)
Stimulation level	Adjustable up to the maximum tolerable intensity according to individual settings
Application site	Vastus lateralis of the thigh

**Table 4:** Average stimulation intensity settings by electrode type (relative units on EMS controller, based on total output of 100 units).

Material	P1 Current Intensity		P2 Current Intensity	
	Total	By Muscle Group	Total	By Muscle Group
A (Lock Stitch)	100	55	100	35
B (Knit)	100	45	100	28
C (Hot Stamping)	100	100	100	100
D (Moss Stitch)	100	50	100	37

## Data Availability

The original contributions presented in this study are included in the article. Further inquiries can be directed to the corresponding authors.
